# Surveillance of the Efficacy of Artemisinin–Piperaquine in the Treatment of Uncomplicated *Plasmodium falciparum* Malaria Among Children Under 5 Years of Age in Est-Mono District, Togo, in 2017

**DOI:** 10.3389/fphar.2020.00784

**Published:** 2020-06-05

**Authors:** Qi Wang, Zhenyan Zhang, Weisheng Yu, Chenguang Lu, Guoming Li, Ziyi Pan, Hongying Zhang, Wanting Wu, Tinah Atcha Oubou, Yueming Yuan, Jiawen Guo, Yuan Liang, Xinan Huang, Wenfeng Guo, Changqing Li, `Nadia Julie, Qin Xu, Logte Sanwogou, Jianping Song, Changsheng Deng

**Affiliations:** ^1^Artemisinin Research Center, Guangzhou University of Chinese Medicine, Guangzhou, China; ^2^Tinah ATCHA OUBOU, National Malaria Control Program, Ministry of Health and Social Security, Lome, Togo; ^3^Institute of Science and Technology, Guangzhou University of Chinese Medicine, Guangzhou, China; ^4^Logte SANWOGOU, Health Bureau, East Mono District, Togo

**Keywords:** artemisinin–piperaquine, *Plasmodium falciparum*, *merozoite surface protein-2*, Togo, gametophyte

## Abstract

**Background:**

Malaria is a major public health concern in Togo. The Est-Mono district of Togo has a population of 150,000. Accordingly, the Guangzhou University of Chinese Medicine, China and the Ministry of Health and Social Security, Togo launched a nationwide Mass Drug Administration Project with artemisinin–piperaquine (AP) in Est-Mono. Before launching this project, the sensitivity test of AP was conducted in a general clinic in Elawagnon, Togo. With this background, we evaluated the efficacy and safety of AP for the treatment of uncomplicated falciparum malaria in children under the age of 5 years.

**Methods:**

Children aged 6–59 months with uncomplicated falciparum malaria were enrolled in this study. The selected patients were treated with a combination regime of artemisinin–piperaquine. The patients were followed up for 28 days, during which signs of the following were observed for: the duration for fever clearance, parasitemia density, gametophyte generation, cure rate, hemoglobin level, and *merozoite surface protein-2* (*msp-2)* polymorphism. The primary end point was a 28-day cure rate and polymerase chain reaction (PCR)-corrected reinfection and recrudescence. This research followed the standardized World Health Organization (WHO) protocol for the assessment of the efficacy of antimalarial drugs.

**Results:**

A total of 91 children with uncomplicated falciparum malaria were enrolled in this study. Adequate clinical and parasitological responses (ACPRs) before and after PCR-correction were 66 (73%) and 90 (99%), respectively. The average hemoglobin level in the patient increased by 0.05 g/dl per day (p < 0.0001) after the treatment. The gametophyte generation did not decline at the beginning of the treatment; however, after 14 days, it declined (day 21: p < 0.05; day 28: p < 0.01). In the *msp-2* polymorphism study of 24 children treated for parasite infection, one case of *msp-2* with *3D7* haplotype and *FC27* haplotype was noted, indicating its recrudescence, with a frequency of 4%. The remaining 23 cases could have been of reinfection, with a frequency of 96%. No serious adverse reactions occurred, and AP was well-tolerated by all patients.

**Conclusion:**

Artemisinin–piperaquine was found to be an effective combination for treating uncomplicated falciparum malaria in children aged <5 years in Togo, and the drugs were well-tolerated. In Togo, *Plasmodium falciparum* remains sensitive to artemisinin–piperaquine, necessitating its trial in this region.

**Clinical Trial Registration:**

Trial registration: ECGPHCM No. B2017-054-01; MHSST AVIS N° 0001/2016/CBRS du 07 janvier 2016. Registered 17 March 2014, http://www.chinadrugtrials.org.cn/eap/main.

## Introduction

Malaria remains a major public health concern in Togo (Dorkenoo et al., 2012; Klement et al., 2014). In 2018, 7,889,095 people were at risk for malaria. The same year, 2,108,823 (median) malaria cases were reported, of which 1,090,110 were of falciparum malaria and 5,132 (median) of malarial deaths (World Health Organization (WHO, 2019)). According to the Togo 2014 National Data for the Plateaux region, malaria accounted for 51% of all outpatient cases and 29% of inpatient cases in medical institutions, with children under the age of 5 years accounting for 37% of all malarial incidences and 29% of all mortality cases (Djadou et al., 2006; Djadou et al., 2007). In Togo, > 10% of the children experiencing fever took the aid of informal private care centers, while most remained untreated, which led to a mortality rate of 8.2% (Assimadi et al., 1998; Dorkenoo et al., 2013; WHO, 2019).

The Ministry of Health and Social Security of Togo (MHSST) decided to implement chemoprophylactic measures for handling seasonal malaria in children under the age of 5 years in the Grassland, Kara, and Central regions (Guidelines for the Treatment of Malaria, 3rd edn (WHO, 2015)). In 2015, the Guangzhou University of Chinese Medicine (GUCM), China, signed an agreement with the MHSST to launch a mass drug administration (MDA) program with artemisinin–piperaquine (AP) for approximately 1.5 million people in the Plateaux region and selected approximately 150,000 people in the Est-Mono province as the pilot study area. AP was introduced as an option to treat uncomplicated falciparum malaria by the National Malaria Control Programme (NMCP) and administrated to Est-Mono inhabitants as the above mentioned MDA program. The latest clinical studies have confirmed >97% efficacy and better compliance in treating uncomplicated falciparum malaria post the program (Srivicha et al., 2007; Song et al., 2008; Nguyen et al., 2012). Meanwhile, the MDA program with AP has been implemented with positive outcomes in Cambodia (Song et al., 2011) and the Comoros (Bo et al., 2015; Bo et al., 2016; Chang et al., 2018), where malarial morbidity and mortality has dropped by >90% after the implementation of the AP-MDA program.

The efficacy and safety of AP have not yet been tested in children under the age of 5 years in Togo. Therefore, sensitivity test for AP was conducted at a general clinic in the Elawagnon county before launching the MDA program universally. With this background, we evaluated the efficacy and safety of AP for the treatment of uncomplicated falciparum malaria in children under the age of 5 years in Elawagnon, Prefecture on Est-Mono province, Togo.

## Methods

### Study Sites

The Est-Mono district covers an area of 2,474 km^2^ in Togo and has a population of 89,060, with a population growth rate of 1.03% in 2010 (Essoya et al., 2012). Est-Mono is one of the nine health districts in the Plateaux region—which is one of the Togo's six such regions. This district is situated in central Togo and has 17 health facilities staffed by nurses, auxiliary nurses, or nonqualified nurses. Each health unit has community health workers (CHWs) trained in health promotion and the management of uncomplicated malaria cases within their communities.

The Est-Mono district (Essoya et al., 2012) and the other central and southern parts of Togo have two rainy seasons in a year, one during April–July and the other during September–November. This district receives an average of 949 mm (± 37.4) annual or 79 mm (± 3.1) monthly precipitation. The climate in Togo is generally tropical, with the average temperatures ranging from 27.5°C (81.5°F) on the coastal areas to approximately 30°C (86°F) in the north. Malarial transmission here is seasonal, with peaks occurring during the rainy seasons. Two villages, EPT and RCPA, in the Elawagnon Prefecture on Est-Mono province were selected as the study area. The time span of the MDA was from March to May 2017.

### Study Design

The study for evaluating suitability of AP in the treatment of uncomplicated falciparum malaria followed drug sensitivity observation, as suggested by the WHO, for 28 days (Methods for surveillance of antimalarial drug efficacy (WHO, 2015), Methods and techniques for assessing exposure to antimalarial drugs in clinical field studies (WHO, 2011)). Based on past researches, the proportion of failed treatments for AP was estimated to be 5%. To ensure that 95% of the cases were treated successfully and that approximately 5% of the patients could not participate in this study, at least 73 people were selected as a sample size at the study site. The calculation was considered under the hypothesis that if 10% of the patients were lost to follow-up and hence excluded, the final sample size would be 80 (Methods for surveillance of antimalarial drug efficacy (WHO, 2015), Methods and techniques for assessing exposure to antimalarial drugs in clinical field studies (WHO, 2008; WHO, 2011)). Therefore, at least 80 participants were considered to be included as the target population for the study site to minimize loss of patient participation.

### Inclusion Criteria

Children aged 6–59 months with body temperature ≥37.5°C, no signs or symptoms of severe malaria, and without obvious symptoms of fever or other symptoms of severe malnutrition (*e.g.*, upper arm length <11 cm, height and weight ratio <70%) were included in the study. Moreover, the participants should be with a single infection caused by *Plasmodium falciparum* parasite at densities of 2,000–250,000/μl and with the ability of intaking oral drugs. Informed consent of the patient's parent or legal guardian was obtained after confirming their convenience to attend clinic examinations and confirming no history/allergic reaction/contraindications to AP in the patients.

### Medicine and Administration

AP tablets (containing 62.5 mg of artemisinin and 375 mg of piperaquine per tablet, Artequick^®^, Artepharm, Co., Ltd., batch number: 20160601) were administered in 1/2 tablet dosage at every 24 h over 2 days for children aged 6–24 months and 3/4 tablet dosage at every 24 h over 2 days for children aged 25–59 months. The medicines were administered under adult supervision. All study participants were provided with acetaminophen treatment every 6 h until the fever symptoms resolved completely.

### Laboratory Inspection

Blood samples were collected from the finger tips on days 0, 2, 3, 7, 14, 21, and 28 to prepare thin and thick blood specimens. The number of asexual bodies/cubic millimeter of the blood sample was calculated before treatment. No asexual protozoa or parasite were detected in 500 fields of thick blood membrane (Methods for surveillance of antimalarial drug efficacy (WHO, 2015)). The hemoglobin level was measured on days 0, 7, 14, 21, and 28 on the Hemoglobin Analyzer URIT-12 (Labon Medical Equipment, China). Blood filter paper was prepared on days 0, 7, 14, 21, and 28, and thin and thick blood glass as well as blood filter paper specimens were made for laboratory testing for patients positive for the parasite. Meanwhile, approximately 3 ml of the venous blood samples was collected on days 0 and 28 from positive patients using heparin vacuum tubes, and the samples were stored at −20°C in the laboratory for white blood cells (WBC) and PCR analysis of polymorphism of *merozoite surface protein-2 (msp-2)* of *P. falciparum* in order to distinguish between incidence of recrudescence and reinfection (Adithya et al., 2003; Aubouy et al., 2003; Basco et al., 2004; Dolmazon et al., 2008; Awaga et al., 2012).

### Outcome Evaluation

Clinical outcomes were evaluated with reference to the classification system suggested by the WHO (Methods for surveillance of antimalarial drug efficacy (WHO, 2015), as follows:

#### Early Treatment Failure (ETF)

Danger signs or severe malaria on days 1, 2, or 3 in the presence of parasitemia; parasitemia on day 2 being higher than that on day 0, irrespective of the axillary temperature; parasitemia on day 3 with axillary temperature of ≥37.5°C; and parasitemia on day 3 of ≥25% of the count on day 0.

#### Late Clinical Failure (LCF)

Danger signs or severe malaria in the presence of parasitemia on any day between days 4 and 28 in patients who did not previously meet any of the criteria of ETF; and presence of parasitemia on any day between days 4 and 28 with axillary temperature of ≥37.5°C in patients who did not previously meet any of the criteria of ETF.

#### Late Parasitological Failure (LPF)

Presence of parasitemia on any day between days 7 and 28 with axillary temperature of <37.5°C in patients who did not previously meet any of the criteria of ETF or LCF.

#### Adequate Clinical and Parasitological Response (ACPR)

Absence of parasitemia on day 28, irrespective of the axillary temperature, in patients who did not previously meet any of the criteria of ETF, LCF, or LPF.

After every administration, the following question was asked: whether there were any adverse reactions, such as mental disorder, insomnia, headache, tinnitus, deafness, nausea, vomiting, loss of appetite, abdominal pain, diarrhea, itching, rash, or other symptoms, and detailed records were made according to the observation of adverse reactions.

### Statistical Analysis

All data was entered twice into Microsoft Excel spreadsheet. The per-protocol (PP) analysis was performed as the primary end-point. The PP population included all participants whose primary endpoint data was at day 28, who received a full course of AP, and who adhered to the follow-up visit schedule.

The GraphPad PRISM 7.0 (GraphPad Software, USA) was employed for all statistical analyses. The Shapiro–Wilk normality test was used to test the normality of the data. For normally distributed data, t-test was used for comparison between two groups in combination with Welch's t-test; and one-way analysis of variance (ANOVA) and Tukey's test were used for multiple comparisons among multiple groups. For nonparametric data, Mann–Whitney test was employed to compare between two groups, while Kruskal–Wallis and Dunn test were used to compare among multiple groups. Bidirectional ANOVA was employed for two-dimensional grouping data. Additionally, parasite clearance time and fever clearance time were represented by the mean ± standard error of the mean (SEM).

## Results

### Study Profile and Baseline Characteristics

**Figure 1:**
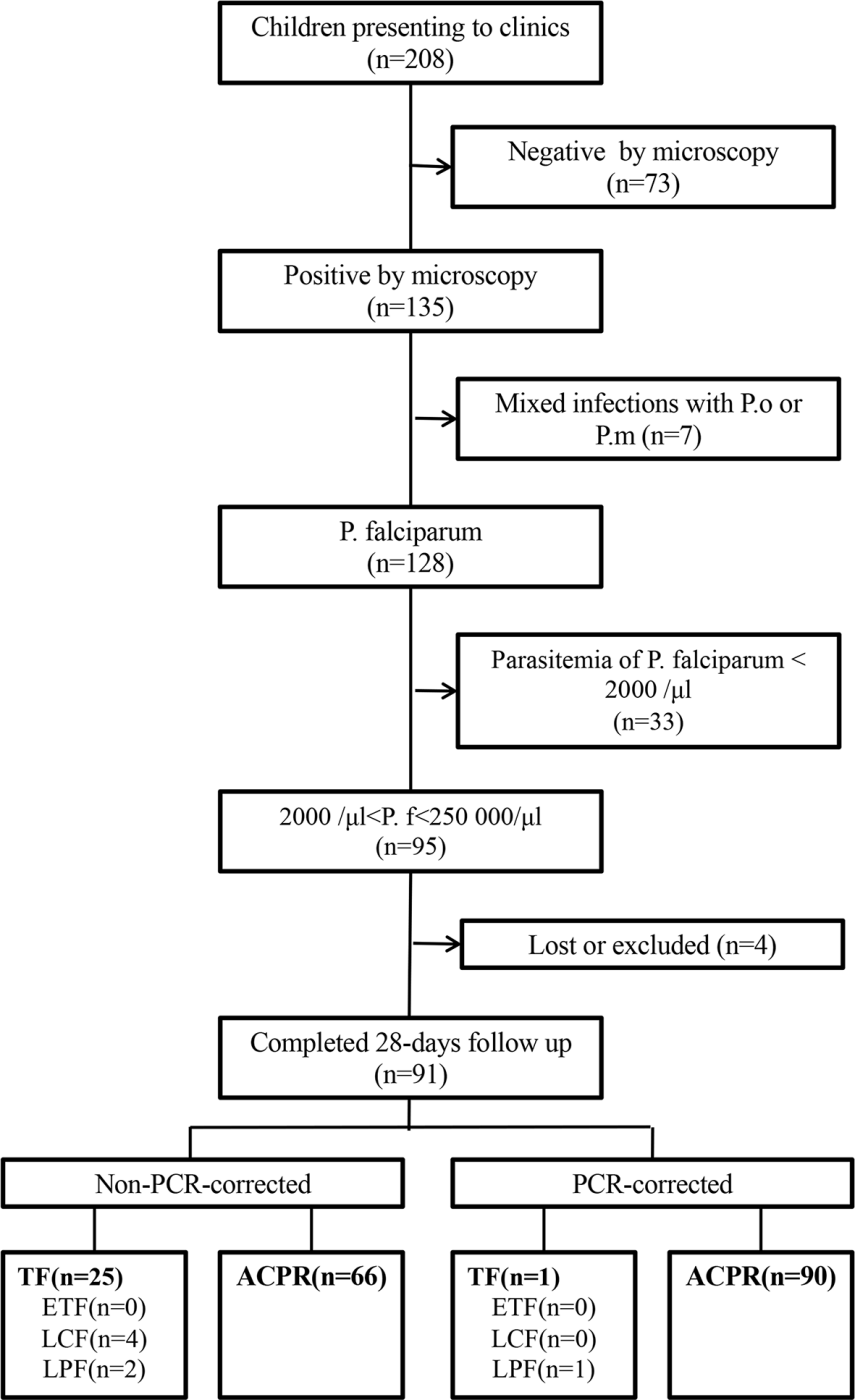
Patient flow chart in the clinical trial. ACPR, adequate clinical and parasitological response; ETF, early treatment failure; LCF, late clinical failure; LPF, late parasitological failure; TF, treatment failure; *P.f*, *Plasmodium falciparum*; *P.m*, *Plasmodium malariae*; *P.o*, *Plasmodium ovale*.

Among 208 children aged 6–59 months who were admitted to the general hospital and presented with fever, 73 (35%) tested negative for malaria by microscopy, 7 (3%) tested positive for mixed infections by *P. ovale* and *P. malariae*, 33 (16%) tested positive for parasitemia with density <2,000 parasites/μl, two (1%) could not fulfill the 28-day follow-up, one (0.5%) received other antimalarial treatment on the first day of the study duration, and one (0.5%) was lost to follow-up. Finally, 91 (44%) patients who met the inclusion criteria were included in this study (**Figure 1**).

The baseline clinical and laboratory characteristics of the study subjects are shown in **Table 1**. The patients (n = 91) had an average age of 31.41 months (age range: 6–59 months) and included 44 (48%) girls. Their average weight was 11.73 kg (weight range: 8.73–14.73 kg), and the average body temperature was 38.5°C (body temperature range: 37.6–39.4°C). Their mean hemoglobin level before treatment was 8.2 g/dl (hemoglobin range: 6.6–9.8 g/dl), and the geometric mean of parasitemia was 36,184 (95% CI: 28,249–44,118).

**Table 1:** Baseline characteristics of the study participants with uncomplicated *Plasmodium falciparum* malaria treated with artemisinin–piperaquine in Togo.

Variable	Enrolled (N = 91)
Age (months), median	31.41(6–59)
Female, %	44(48%)
Weight, mean (kg)	11.73(8.73–14.73)
Temperature at enrollment, median (°C)	38.5(37.6–39.4)
Pretreatment hemoglobin concentration, median (g/dl)	8.2(6.6–9.8)
Parasitemia, parasites/µl, 95%CI	36,184(28,249–44,118)

### Parasitemia and Fever Clearance Time

**Figure 2:**
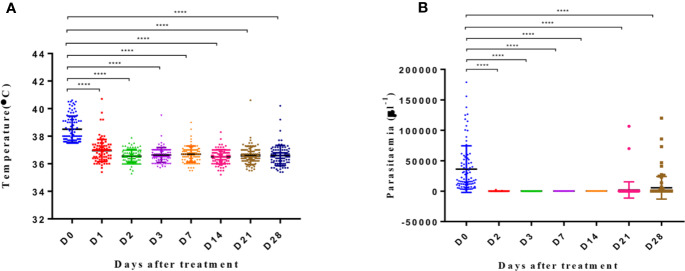
Body temperature and parasitemia across days after treatment. Y axis represents the temperature **(A)** and parasitemia **(B)** in patients. The significance levels are shown as significant (****: p < 0.0001) and nonsignificant (NS). All data were analyzed with one-way ANOVA and multiple comparisons. Error bars show the standard error of the mean (SEM).

After AP treatment, most patients experienced rapid remission of clinical symptoms. For instance, their average body temperature of 38.5°C on day 0 fell to 36.5°C on day 2 (p < 0.0001), with 79 (87%) patients reporting a temperature of <37°C (**Figure 2A**). On day 2 of treatment, 66 (73%) patients showed clearance of parasites in their blood smears, with complete clearance observed on day 3 (**Figure 2B**).

### Gametophyte and Hematological Parameters

**Figure 3:**
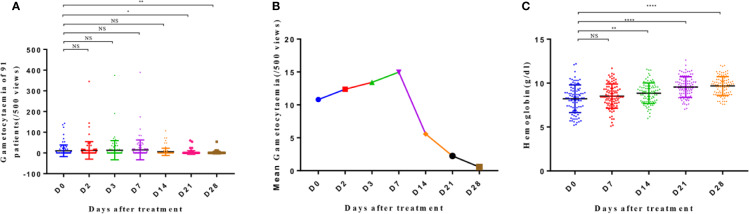
Gametocytemia **(A)**, mean gametocytemia **(B)**, and hemoglobin level **(C)** of patients from day 0 to day 28. The significance levels are shown as significant (****: p < 0.0001; **: p < 0. 01; *: p < 0. 05) and nonsignificant (NS). The gametocytemia and hemoglobin level of different groups were analyzed with one-way ANOVA and multiple comparisons. Error bars show the standard error of the mean (SEM).

The gametophyte observation (**Figures 3A, B**) revealed that the average gametocytemia increased until day 7, and the number of patients with gametophyte did not decrease significantly. Both these parameters decreased synchronously after day 7 of treatment, with significant reduction noted on day 21 (p < 0.05) and day 28 (p < 0.01). Gametophytes were continually observed on blood smears until day 28 in six patients, with a mean value of 8/µl.

The hemoglobin level of the patients improved progressively from day 0 to day 14 and on days 21 and 28 (**Figure 3C**). Significant differences were noted in the hemoglobin levels on days 14, 21, and 28 when compared to that on day 0 (p < 0.01; p < 0.0001; p < 0.0001, respectively), with an average increase in the level of 0.05 g/dl per day.

### Cure Rates

In the PP analyses, PCR-corrected ACPR was found to be 90 (99%) for AP (**Table 2**), and treatment failure was only one for recrudescence. On the other hand, non-PCR-corrected ACPR was 66 (73%), including four (4%) for LCF and 21 (23%) for LPF.

**Table 2:** Efficacy outcomes and adequate clinical and parasitological response rates of artemisinin–piperaquine treatment by day 28 among the participants.

Variable	Enrolled (N = 91)
Late parasitological failure-no (%)	27%
Recrudescence	1
Reinfection	24
Indeterminate or sample unavailable	0
PCR-uncorrected cure rate-% (95%CI)	
ACPR(%)	66(73%)
ETF	0
LCF	4
LPF	21
TF(%)	25(27%)
PCR-corrected cure rate-%(95%CI)	
ACPR(%)	90(99%)
ETF	0
LCF	0
LPF	1
TF(%)	1(1%)
Parasite clearance time, mean ± SD hours*	57.3 ± 14.7
Fever clearance time, mean ± SD hours*	32.4 ± 16.2

*Parasite clearance time and fever clearance time were represented by the mean ± standard error of the mean (SEM).

### Polymorphism of *msp-2* and Clinical Parameters of 24 Positive Patients

Two allele genes (*3D7* and *FC27*) were detected in 24 patients who remained positive for malaria despite AP treatment by nested PCR. In these patients, *msp-2* of all isolates was analyzed before and after treatment. The condition of only one patient, whose 3D7 and FC27 haplotypes of *msp-2* were the same before and after taking the drugs, suggested recrudescence, with a frequency of 4% (1/24). The remaining 23 patients may have been reinfected, with a frequency of 96% (23/24).

**Figure 4:**
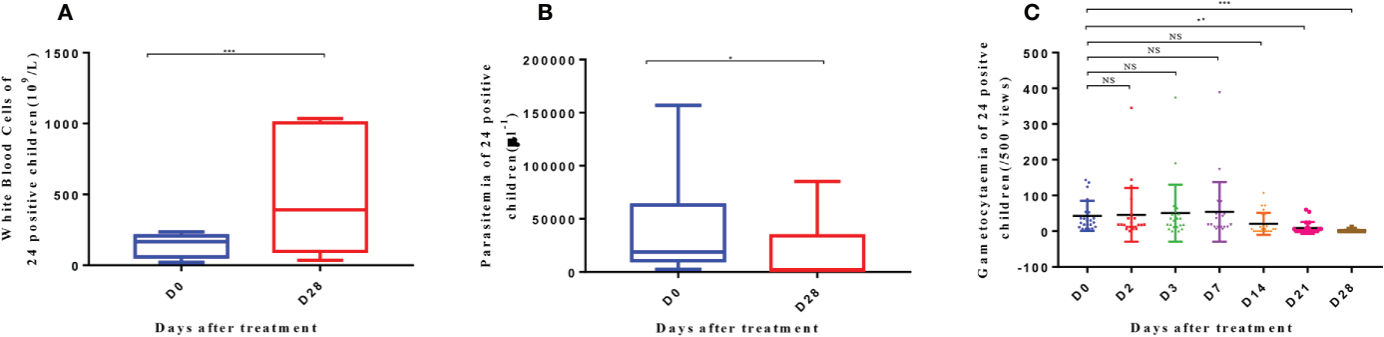
White blood cells **(A)**, parasitemia **(B)**, and gametocytemia **(C)** of 24 patients with positive parasite detection after treatment on D0 and D28. The significance levels are shown as: significant (***: p < 0. 001; **: p < 0. 01; *: p < 0. 05) and nonsignificant (NS). White blood cells and parasitemia of 24 patients were analyzed with one-way ANOVA, and gametocytemia was analyzed with one-way ANOVA and multiple comparisons. Error bars show the standard error of the mean (SEM).

Meanwhile, the WBC levels of the 24 positive patients increased on day 28 after AP treatment (p < 0.001) (**Figure 4A**). The parasitemia density in these patients also decreased (p < 0.05) (**Figure 4B**). Moreover, the generation of gametophytes decreased from day 21 (p < 0.01) to day 28 (p < 0.001) (**Figure 4C**).

### Adverse Reactions

In this study, the most common adverse reaction recorded was cough, followed by diarrhea and rash in all 91 patients who undertook AP treatment. Other notable adverse reactions included loss of appetite, vomiting, and abdominal pain. No serious adverse reactions occurred, and AP seemed to be well tolerated. The list of adverse reactions in the patients after the treatment is given in **Table 3**.

**Table 3:** Summary of adverse reactions of patients with uncomplicated falciparum malaria treated with artemisinin–piperaquine combination regime in Togo.

Adverse events	AP, N(%)
Cough	6 (6)
Diarrhea	1 (1)
Loss of appetite	1 (1)
Vomiting	2 (2)
Abdominal pain	2 (2)
Insomnia	0
Irritability	0
Headache	1 (1)
Rash	1 (1)

## Discussion

Malaria is the leading public health concern in Togo. This disease is prevalent throughout the country and strikes in all 12 months of the year. Moreover, the outbreak often occurs in the rainy season. According to the National Health Authority data of Togo in 2014, approximately 11 million malaria cases occur every year in Togo, with children aged <5 years being the most affected, accounting for 60.5% of all cases (Awag et al., 2008). In a secondary data analyses from the regional health information system on confirmed and suspected malaria cases, an annual record of 114,654 malaria cases (19,109 ± 6,622) was reported between January 2005 and December 2010, with the number of cases increasing from 10,299 in 2005 to 26,678 in 2010 (p < 0.001). The prevalence of confirmed malaria cases increased from 23.1‰ in 2005 to 257.5‰ in 2010 (p < 0.001). Despite the implementation of the artemisinin combination therapies (ACTs) and CHWs strategies in Togo, the study demonstrated an increase in the prevalence of malaria.

A few studies have reported the efficacy of malarial treatment and resistance to ACTs in Togo (Djadou et al., 2007; Dorkenoo et al., 2012; Améyo et al., 2016.). Only one article reported about the effect of ACTs (Dorkenoo et al., 2012). The therapeutic effects of artemether–benflumetol (AL) and artesunate–amodiaquine (ASAQ) were monitored in the National Malaria Control Program (NMCP) of Togo in 2013. In this program, a PCR-corrected ACPR of 97–100% was suggested for AL and of 96.3–100% for ASAQ. However, the recorded adverse events were significant: 2.68% for AL and 1.53% for ASAQ. In Benin, a country bordering Togo, a 42-day AL-efficacy study was conducted in 2014 (Aurore et al., 2016). On day 1 of the treatment in their study, the author reported apparent clearance of fever, with a clearance rate of parasites of approximately 90%; on day 2, almost all parasites were cleared, with the hemoglobin level rising slightly in sync with increasing clearance rate of the parasite. In their study, the non-PCR-corrected ACPR was 75.6% after 42-day follow-up, while PCR-corrected ACPR was 100%. These observations indicated that all treatment failures occurred due to refection, which supports the findings of the two previously mentioned papers and the present one (Dorkenoo et al., 2012; Aurore et al., 2016). The WHO recommends changing of the treatment policies only when the PCR-corrected treatment failure is >10%. As this was not the case in Togo, ACTs remain sensitive here.

During the sensitivity test of AP in this paper, fever was observed to reduce in most participating patients on day 1, with only one patient recording a temperature >37.5°C on day 2, and complete parasite clearance was observed on day 3. In addition, the average gametophyte generation increased, and the number of gametophyte cases did not reduce significantly in the first 7 days of the treatment. However, until day 28, the average gametophyte generation rapidly declined, probably because AP only affected parasites in the blood stage and could not directly kill the gametophytes already present. As a result, no obvious change was recorded in the early performance for patients carrying the gametophytes, although mature gametes were produced in some patients. Later, in the blood stage, a large number of parasites were killed, which led to a sharp decline in the number of late mature gametophytes; to a certain extent, this phenomenon inhibited the transmission of parasites by mosquitoes (Sophie et al., 2011). Overall, with respect to parasite clearance reduction, the reduction in gametophytic carrier rate was consistent with the improvements in the hemoglobin levels in all 91 participating patients. In our study, after PCR proofreading genotyping, the efficacy threshold of AP reached 98.90%, which is greater than the 95% efficacy threshold recommended by the WHO for AL (Guidelines for the treatment of malaria (WHO, 2010)). In addition, AP was well-tolerated by the local children in Togo, and no serious or adverse events, including diarrhea, weakness, anorexia, hemoglobinuria, itching, or eyelid edema, were noted during the 28-day follow-up in this study.

Our results conform to those of ACT studies conducted over the past 5 years in the Sub-Saharan Africa. The ACT combination therapy has a high cure rate for falciparum malaria without any complications. A past randomized study (Bernhards et al., 2014) conducted in Kenya in 2014 reported PCR-corrected ACPR of 97.8% for AL and 99.1% for dihydroartemisinin–piperaquine (DP). In addition, a single-arm prospective study (Busiku et al., 2014) conducted in Zambia in 2012 showed that 98% of the participating patients achieved complete clearance of their parasitic diseases on day 3, with PCR-corrected ACPR of 100% for AL. In other words, all participating patients achieved ACPR. In another study (Mateusz et al., 2017) conducted in three provinces of Angola in 2015, the 28-day cure rate of AL after PCR-correction was 88.1–96.3%. No evidence of artemisinin resistance was recorded in this study. However, the *pfmdr1* haplotype was detected in all cases of AL treatment failures, indicating its association with decreased sensitivity of phenylfluorene. Another study (Magdalena et al., 2014) conducted in Malawi in 2014 reported a non-PCR-corrected cure rate for AL of 69–82.5%, but the PCR-corrected APCR was 98–100%, which was associated with high reinfection. These authors attributed this phenomenon to the short half-life of phenylfluorene (*i.e.*, 3–6 days). Some researchers (Stephen et al., 2014) evaluated the efficacy of ACT in six geographic regions of Nigeria and found that AL and ASAQ were highly effective in Nigeria, with the PCR-corrected cure rates of 96.9 and 98.3%, respectively.

## Conclusion

AP was determined to be effective in treating uncomplicated falciparum malaria and was well-tolerated in affected children under the age of 5 years in Elawagnon, Prefecture on East-Mono province, Togo. Hence, it was confirmed that *P. falciparum* remains sensitive to AP in Togo, which could be further tested *via* extensive drug trial in this region.

## Data Availability Statement

All datasets generated for this study are included in the article/supplementary material.

## Ethics Statement

The studies involving human participants were reviewed and approved by Trial registration: ECGPHCM No. B2017-054-01; MHSST AVIS N° 0001/2016/CBRS du 07 janvier 2016. Registered 17 March 2014, http://www.chinadrugtrials.org.cn/eap/main. Written informed consent to participate in this study was provided by the participants' legal guardian/next of kin.

## Author Contributions

QW performed the statistical analysis and drafted the manuscript. CLu, WY, ZZ, WW, GL, YL, and GL collected and interpreted the data. HZ and ZP conducted the research and performed the PCR analysis. XH, WG, CLi, MC, JG, TO, LS, and QX collected the data and conducted the research. JS conceived and designed the study, conducted the research. CD conducted the research. All authors read and approved the final manuscript.

## Funding

This work was supported by Guangzhou Science and Technology Program [Grant Number 201807010007] to JS and QW, China post-doctoral science foundation[Grant Number A2-2902-19-414-007] to QW, Natural Science Foundation of China [Grant Number 81873218] to JS and QW, National Major Science and Technology Projects of China (CN) [Grant Number 2018ZX09303008] to CD and QW, Project of Traditional Chinese Medicine Bureau of Guangdong [Grant Number 2019(43)] to JS and QW, and YangFan Innovative And Entrepreneurial Research Team Project[Grant Number 2014YT02S008] to CD and QW.

## Conflict of Interest

The authors declare that the research was conducted in the absence of any commercial or financial relationships that could be construed as a potential conflict of interest.

## Abbreviations

ETF, Early treatment failure; ACPR, Adequate clinical and parasitological response; ACT, Artemisinin-based combination therapy; LCF, Late clinical failure; LPF, Late parasitological failure; NMCP, National Malaria Control Programme; WHO, World Health Organization; DP, Dihydroartemisinin-piperaquine; AL, Artemether-benflumetol; ASAQ, Artesunate-amodiaquine; AP, Artemisinin piperaquine.
